# A statistical mechanical problem?

**DOI:** 10.3389/fpsyg.2014.00947

**Published:** 2014-09-02

**Authors:** Tommaso Costa, Mario Ferraro

**Affiliations:** ^1^Department of Psychology, University of TurinTurin, Italy; ^2^Department of Physics, University of TurinTurin, Italy

**Keywords:** neural network, statistichal mechanics, behavior, connectivity, mapping

## Abstract

The problem of deriving the processes of perception and cognition or the modes of behavior from states of the brain appears to be unsolvable in view of the huge numbers of elements involved. However, neural activities are not random, nor independent, but constrained to form spatio-temporal patterns, and thanks to these restrictions, which in turn are due to connections among neurons, the problem can at least be approached. The situation is similar to what happens in large physical ensembles, where global behaviors are derived by microscopic properties. Despite the obvious differences between neural and physical systems a statistical mechanics approach is almost inescapable, since dynamics of the brain as a whole are clearly determined by the outputs of single neurons. In this paper it will be shown how, starting from very simple systems, connectivity engenders levels of increasing complexity in the functions of the brain depending on specific constraints. Correspondingly levels of explanations must take into account the fundamental role of constraints and assign at each level proper model structures and variables, that, on one hand, emerge from outputs of the lower levels, and yet are specific, in that they ignore irrelevant details.

## 1. Introduction

Any attempt to derive the processes of perception and cognition or the modes of behavior from sets of neural activities is confronted with the problem of mapping an incredibly large set of possible brain states to a very large number of observables. Simply put, the numbers are staggering: although estimates vary, there are purportedly about *N* = 10^11^ neurons in the human brain (Sporns, 2012) and even with the very drastic simplification that a neuron is a binary device, possible states are 2^*N*^ = 2^10^11^^. This enormous set of states must be mapped into the possible observables and even in this case numbers are huge: for instance even with a conservative estimate the number of possible postures is 10^30^ (Stephens et al., 2011). The sheer orders of magnitude involved seem to prevent the possibility of finding any correspondence among elements of the two sets, i.e., the matching of states to observable processes.

Fortunately there are factors that somewhat simplify the problem: for instance a given behavior can result from many different brain states, as redundancy is a well known evolutionary feature to make living systems more robust. Furthermore brains are made up of very complex networks (connections are of the order of 10^15^), thus neural states are not independent variables and they tend to form spatio-temporal patterns, rather that disordered sequences of activity. Indeed, fMRI measures have shown that spatial maps of activity are formed even in resting state situations, without any external stimulus (Raichle, 2010). In addition, as suggested in Ganguli and Sompolinsky (2012), states of the dynamical systems describing the activity of cortical areas (e.g., motor cortex, or sensory cortex) are limited by the dimensionality of the inputs (e.g., motor task to be performed, or sensory inputs), which is often much lower than the dimensionality of the cortical dynamical system.

These simplifying factors notwithstanding, the brain is so complex that to explain cognitive and behavioral functions philosophers and scientists have often resorted to conceptual metaphors (Daugman, 1993); modern examples are the computer and information metaphor (see Werner, 2011) for a critical review.

An earlier version of the computation metaphor, based on the seminal work of McCulloch and Pitts (1943), on the equivalence between networks of formal neurons and Turing machines, was centered on the notion that neural activity implements logical calculus via formal rules for the transformation of for the manipulation of symbols (Daugman, 1993), an idea which has provided much impulse to the development of artificial neural networks and their applications (Haykin, 1994; Werner, 2011). The computation metaphor later has given rise to the so called “computational theory” of the brain whose aim is to explain and to simulate the mechanisms by which the brain performs a variety of tasks such as, for instance, edge detection or stereo vision (Marr, 1982). This version of the computation metaphor has became so popular that the term “computational” is nowadays used to characterize almost any model including task analysis (Daugman, 1993).

Complementary to this approach is the information metaphor, that views the brain as an information processing device and focuses on the input–output relations among neurons in the framework of information theory. The central issues in this framework are those of coding and decoding of the neural stimulus, namely which feature of a neural spike train (rate, correlations, etc.) carries the information (in Shannon's sense) and, next, how this information is decoded by the brain, revealing the nature of the external (physical) stimulus (Jacobs et al., 2009; Werner, 2011). The latter problem is known to be an inference problem (Knill and Pouget, 2004), to solve which Bayesian techniques have proven to be very successful. This has lead to the “Bayesian coding hypothesis”: the brain represents sensory information in the form of probabilities and derives posterior probabilities of the configurations of the external world (Knill and Pouget, 2004; Doya, 2007; Friston, 2012).

Computation and information metaphors are useful to elucidate important aspects of brain function, but, as pointed out in Werner (2011), they fail to provide the fundamental link between the dynamics of neural activity and computational and information processing properties of the brain. Thus, a different approach has emerged which maintains that real comprehension of cognitive and behavioral functions can only follow from the analysis and explanation of the collective dynamics of neural systems (Werner, 2011; Parker and Srivastava, 2013).

This is also the point of view taken in the present work: specific models related to this approach will be reviewed in more detail later.

Neuronal activity takes place at different scales and a rough classification can distinguish between microscopic (neurons and synapses), mesoscopic (networks and local interactions between neurons), and macroscopic levels (areas of the brain) (Deco et al., 2008). All these levels have their own specificity determined by different types of activity patterns. Then understanding the dynamics of the nervous system requires insights into processes occurring at different scales and that must be matched by appropriate levels of description or representation, characterized by specific variables and model structures.

Different neural models can be represented as elements of a two dimensional space (Cessac and Samuelides, 2007). The first axis of this space describes the type of neuron and its proximity to biology, starting from the Hodgkin–Huxley equations followed by excitable systems with continuous state and finally binary neurons of the McCulloch–Pitts type. The other axis takes in account the collective aspect of neural networks in a hierarchy of ordering: one neuron, few neurons, one population of weakly coupled neurons and finally one population with arbitrary coupling.

Large neural populations present an obvious similarity with physical systems composed of very large number of elements (atoms or molecules) subjected to mutual interactions. In physics the answer to challenges posed by such systems is to resort to mechanical statistical methods, which do not try to solve models at the microscopical level of individual elements, but, instead, use laws of probability to derive a set of collective variables, whose properties can then be studied at the macroscopic level. The success of this approach requires, and indeed depends on, finding the right variables, which can lead to meaningful macroscopic representations, while disregarding irrelevant ones. This, in turn, involves simplifying the system under consideration, from a detailed description to a more abstract representation in which some properties of the elements forming the system are disregarded.

It must be kept in mind, however, there are crucial differences when considering physical vs. neurobiological systems.

1. First, neural systems of the brain are part of living organisms. The problems concerning the transitions from inert to living states of matter and the characterization of life (Smith and Szathmary, 1997; Longo and Montévil, 2012) are outside the scope of this paper. It is enough to say that, at a fundamental level, activity of neural systems is constrained by the amount of metabolic energy available and by the need to limit entropy production (Schrödinger, 1956; Longo and Montévil, 2012). More relevant for our work is the fact that animal brains have been shaped by evolutionary pressures and, therefore, neural systems are subjected to many cost-benefits trade-offs, the most basic involving the balance between the speed of respose against the accuracy of identification of a stimulus (Geary, 2005).
  These constraints affect the topology of the connections: empirical evidence suggests that brain anatomical connectivity is locally clustered with a few long-range connections between any pair of regions, and this can be explained by the need to minimize wiring costs while maintaining the possibility of long range interactions among different areas (Bassett and Bullmore, 2006).
2. Neurons interact with the rest of the organism and among themselves in ways, in general, more complex than interactions among elements of physical systems. Furthermore, neurons are computational units, able to perform non trivial computations (Koch, 2004).
3. Differently from physics where the elements of a system can be considered all equal (“all electron are the same” as Fermi put it), neural systems are characterized by heterogeneity, e.g., excitatory vs. inhibitory neurons or electrical vs. chemical coupling.
4. Neural systems are endowed with specific architectures, gauged to specific sensory, motor, and cognitive tasks.
5. Networks can learn by changing the strength of their mutual connections.
6. In physical systems the global behavior can be represented by simple scalars, for instance critical exponents and correlation lengths in non-equilibrium phase transitions, whereas models of large networks in the brain must explain the complex spatio-temporal patterns that make up physiological or behavioral responses. Therefore the question arises of what constitutes the relevant definition of system activity for a given level of explanation.

These differences notwithstanding, a statistical mechanics approach is almost inescapable, since dynamics of the brain as a whole are obviously determined by patterns of neural activities occurring at a lower level, and, indeed statistical mechanics tries to derive the laws at the macroscopic level from interactions among microscopic components.

A classical example are, in the theory of artificial neural networks, the so called Hopfield networks of binary units, (see Hopfield, 1982; Amit, 1992) and, for more recent results, (Advani et al., 2013).

Statistical mechanical techniques are not restricted to the Hopfield model (Coolen and Del Prete, 2003): they have been applied also to biological neural systems both to explain experimental data (Masoller et al., 2009; Montani et al., 2009; Deco et al., 2012) and to provide general models of the brain (Ingber, 1981; Freeman and Vitiello, 2006; Parker and Srivastava, 2013).

It will be argued here that the problem of modeling and representing neural systems of increasing size and complexity is akin to the problem of statistical mechanics and that the way out of the problem of intractability is the same: to assign at each scale proper variables, namely variables that emerge from outputs of the lower level, while ignoring details which are irrelevant for the higher level.

In particular, the main claims of this paper are:

- Systems at each level obey to the laws holding for the lower levels, but they are subjected to new constraints that in turn generate new features, like novel patterns of activity, requiring adequate levels of representations.
- Constraints derive from the neural connections whose complexity increases with the dimension of neural circuits, whose topology then plays a central role in determining neural dynamics.

This approach is inspired by the ideas of Jacob (1977) on the structure of natural systems:

“*Nature functions by integration…. Each system at a given level uses as ingredients some systems of the simpler level but some only. The hierarchy in the complexity of objects is thus accompanied by a series of restrictions of limitations. At each level new properties may appear that impose new constraint on the system… Those (constraints) that operate at a given levels are still valid at a more complex level*.”

## 2. Levels of complexity and explanation

Levels of explanations are determined by two main issues: the choice of state variables and the formal structure of the model.

In very general terms, a neural network is a dynamical system describing the temporal evolution of the activities, {*a_i_*} *i* = 1, … *n*, of a neural population of *n* elements, and can be formally expressed by a map *ϕ* which starting from the state at initial time *t*_0_ yields the state at time *t*

(1) $$\phi_{i}:a_{i}\left(t_{0}\right)\to a_{i}\left(t\right);$$

this system can be either deterministic or ruled by probabilistic laws. Maps *ϕ_i_* are usually the solutions of systems of differential equations and their formal expressions are typically very complex, as they depend on a set of external inputs {*I_j_*} *j* = 1, … *m* and on the connections among neurons. Thus, in general some simplifications are carried out to make the dynamical system more manageable.

First one must decide which variable represents the neural activity: this choice is important not just in order to simplify the problem but because, implicitly, it identifies which aspect of neuron dynamics is considered to be important.

Usually in neural networks theory the elementary computational element is assumed to be the single neuron and the basic variable is the potential *V* across the membrane, but other, finer, levels of resolution could be considered, for instance ion species and channels or, in principle, the quantum mechanical scale. Suppose, for sake of argument, that it is possible to write down and solve the Schrodinger equation for any molecule or atom of the neuron: the result would be the an incredibly complex wave function which would not explain more than Hodgkin and Huxley theory, because the quantum mechanical scale is not really necessary to understand how spikes are generated, even though, obviously, the laws of quantum mechanics apply to all atoms forming the neuron.

In conclusion, for a neuron a “natural” variable is the difference of potential *V* across the membrane, whose dynamics are formally described by the theory of Hodgkin and Huxley. However, the level of detail of this model is not really required when one moves from single neurons to neural networks and more abstract models can be developed, whose structure implicitly defines which aspects of spikes generation and transmission are considered important.

For instance, information transmitted along the nervous system of an organism is thought to be encoded by the frequency of the action potentials (the firing rate), and/or by the timing of spikes. Then in modeling the transmission of information one can disregard the shape of the spike and just consider the time intervals with which action potentials occur: this approach is at the basis of the “integrate and fire” type of neuron models (Koch, 2004; Deco et al., 2008).

Neural dynamics can be also described by the temporal variation of spike rates: activity is now identified with the frequency of action potentials and the sequence of spikes collapsed in just one number. The reasons behind this choice, besides the obvious simplification, are based on the observation that many neurobiological phenomena appear to be determined at the level of firing rate. Indeed many experimental data are reported in term of spike rate, which is considered the fundamental element in the information processing in the brain, an idea that goes back to the fundamental work of Adrian (1926). It must be noted that, in recent years, the idea that spike rate suffices to explain coding and decoding of neural signal has been, rather convincingly, questioned (Rieke, 1999).

The rest of this section will try to clarify how increasing complexity of connectivity patterns engenders the emergence of new properties of neural systems and how levels of explanation can be found matching this evolution from simple to complex systems.

### 2.1. Minimal networks

First we shall consider minimal systems of neuron pairs and a rather abstract and very simple version of the Wilson–Cowan model (Wilson, 1999) will be adopted to illustrate the role of the connections in neural dynamics. The state of the neuron will be represented by its activity *a*, a real variable, which evolves according to a set of differential equations. It is not important here to give a precise definition of activity, which can be, for instance, *V* or spike rate, as the equations used in the following can be adapted to different meanings of *a*. Note that the results described in the sequel are general and not depending on the particular form of the equations, used here solely for illustrative purposes.

The activity of a neuron is described by just one differential equation of the form

(2) $$\tau \frac{da}{dt}=-a+S(I-\theta ),$$

where τ is a time constant, *θ* a threshold and *I* the total input that can originate from the external world or, more frequently, from other neurons: in this latter case *I* can be the sum of several inputs. The term −*a* just expresses the obvious idea that in absence of input the activity relaxes to zero, whereas the function *S* defines the effect of the input *I* on the activity *a* of the neuron and it can be modeled in a variety of ways: usually it is assumed *S* to be a monotonically increasing function, with *S(I)* = 0 if *I* − *θ* ≤ 0 and tending to a finite value as *I* increases.

In the following *θ* will be set to 0 and for simplicity's sake it will be supposed that, at least in a time interval δ*t*, *I* is constant. Under these assumptions it is straightforward to show that when the input signal *I* is switched on the activity *a* tends to reach a value a^*^ = *S(I)*. Note that, as *S* is monotonous, there is an one-to-one relation between a^*^ and *I*, so that for any given a^*^ there exists just one value of *I* satisfying the equality a^*^ = *S(I)*; this means that the activity *a* just scale-transforms the input signal, i.e., reproduces *I* on a different scale.

Very different, more complex, activity patterns appear in system of mutually connected neurons, even when just two units are considered: the activity is now a vector

$$a=\left(a_{1},a_{2}\right)$$

and the corresponding system can be written as

(3) $$\begin{array}{l} \tau_{1}\frac{da_{1}}{dt}=-a_{1}+S\left(w_{1}a_{2}+I_{1}\right)\\ \tau_{2}\frac{da_{2}}{dt}=-a_{2}+S\left(w_{2}a_{1}+I_{2}\right) \end{array}$$

The new elements here are the synaptic weights *w*_1_, *w*_2_, that provide a description of the interaction between the neurons: three cases are possible, each characterized by a specific dynamic:

1. the 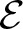 − 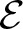 system, where the connections are mutually excitatory, that is *w*_*i*_ > 0, *i* = 1, 2,
2. the 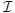 − 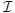 system characterized by mutually inhibitory connections, *w*_*i*_ < 0, *i* = 1, 2,
3. the 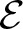 − 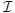 system, where one neuron is excitatory and the other is inhibitory, and the synaptic weights have opposite signs.

If the connections are mutually excitatory the network is a bistable system, namely it is characterized by two stable states (attractors): the activity of both neurons can be either low (possibly zero) or it can reach high activity levels, depending on the values of synaptic weights *w*_1_, *w*_2_, and on the inputs *I*_1_, *I*_2_. This very simple network shows that connections between neurons give rise to a set of new behaviors: for instance, also a very short (ideally instantaneous) stimulus to one of the neurons can trigger the evolution of both neurons toward stable high activity levels, i.e., the system is able to self sustain even when the inputs *I*_1_, *I*_2_ are switched off. Attractors of this system are the simplest instance of multi-stabilities, that can be the basis of short time memory (Wilson, 1999) and can provide a mechanism for the switching between different perceptions or behaviors, as suggested by theoretical and experimental studies, (Deco et al., 2007; Moreno-Bote et al., 2007).

Two mutually inhibitory neurons are an elementary example of winner-takes-all networks, which have been widely used in the context of artificial intelligence and pattern recognition. Due to mutual inhibition one of the two neurons has high activity levels whereas the other is not active. The “winning” neuron is determined by the parameters of the system: in particular if *w*_1_ = *w*_2_ neuron with the larger input “wins.” Such type of network implements the very general principle of competitive exclusion, found also in ecology and population theory, by which when two population compete for resources just one survives (Murray, 2002). Mechanisms of the winner takes all types are thought to be at the basis of selection processes, motor control and path integration (Wilson, 1999).

Finally, a 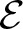 − 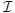 system gives rise to the emergence of homoeostasis mechanisms, by which sensory input is regulated, for instance to make the localization of its sources more precise. Moreover the system can be modified in a straightforward way to produce sustained oscillations also in presence of a constant stimulus, an ubiquitous feature in living organisms, from cardiac cycles to the rhythms of breathing and locomotion. Note that this property is unique for the 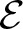 − 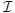 arrangement, in that it is straightforward to show with the standard methods of the theory of dynamical systems that such oscillations cannot appear in either mutually excitatory or mutually inhibitory systems. It follows then that oscillations under constant stimulus are due to the heterogeneity of the system, i.e., the presence of both excitatory and inhibitory connections. Pairs of 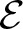 − 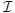 type can in turn be connected into coupled oscillators that act as central pattern generators, controlling motion routines (Kleinfeld and Sompolinsky, 1988; Brunel and Wang, 2001).

In conclusion these simple neural systems show that coupling between neurons gives rise to a variety of activity patterns, more complex than those of a single neuron; hence, they exhibit a larger spectrum of computational and behavioral properties. The basis of this enhanced capability resides in the fact that now *a*_1_, *a*_2_ do not depend solely on the inputs, as connections make them dependent one on the other.

Consider a pair of neurons to which are given inputs *I*_1_, *I*_2_, respectively: if they are not connected the attractors of activity are a^*^_1_ = *S*(*I*_1_), a^*^_2_ = *S*(*I*_2_). As mentioned before there is a one-to-one correspondence between a^*^ and *I* and therefore any activity pair of values a^*^_1_, a^*^_2_ can be reached given suitable inputs *I*_1_, *I*_2_, since activities are independent one from the other. On the contrary mutual dependence of activities limits the number of states the system can reach. Suppose that neurons now form, for instance, a 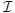 − 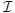 pair. In this case it is straightforward to show that if *w*_1_ = *w*_2_ and *I*_1_ > *I*_2_ the attractors of this system are a^*^_1_ = *S*(*I*_1_), *a*_2_ = 0, that is the second neuron will be inactive whatever be the value of *I*_2_, provided of course that *I*_1_ > *I*_2_.

This idea can be made more precise if one considers activities *a*_*i*_, *i* = 1, 2, as stochastic variables, with randomness due to fluctuations of the stimulus or to stochasticity in the mechanisms generating the neural response.

The probability that *a*_*i*_ take values in a given interval *a*_*i*_ + *da*_*i*_ can be computed, at least in principle, via the Fokker Planck equation (Deco et al., 2008). The derivation of such equation is not trivial and its solution is, usually, very difficult, but some qualitative results can be readily obtained. Let *p*(*a_i_*), *i* = 1, 2, be the probability density functions (pdf) of activities *a_i_* and let *p*(**a**) be the pdf of the activity vector **a**; if neurons are supposed to be independent then the entropy *H*(**a**) of the stochastic variable **a**, a measure of disorder of the system, is the sum of the entropies of the single variables *a*_*i*_, *H*(**a**) = *H*(*a*_1_) + *H*(*a_2_*). On the other hand it is a standard result of probability theory that if *a*_*i*_ are not independent, as in case of connected neurons, *H*(**a**) < *H*(*a*_1_) + *H*(*a*_2_). Thus, connections among neurons reduce the effect of casual fluctuations and this in turn entails the generation of more complex activity patterns.

Mutual dependence of activities has another important consequence: let the activities *a*_*i*_, *i* = 1, 2 be the input of some neuron *j*, and let, for simplicity, assume the weight connecting the input neurons *i* = 1, 2 with *j* to be equal to 1. The total input reaching *j* is *I* = *a*_1_ + *a*_2_ and its variance σ^2^_*I*_ ≤ σ^2^_*a*__1_ + σ^2^_*a*__2_, where the equality holds only if the activities *a*_*j*_ are independent. We see then that mutual connections provide more reliable global inputs.

### 2.2. Large neural systems

It has been shown, so far, that even very simple systems of connected neurons can implement processes of self-organization and entropy reduction. These properties are inherited by large neural populations, but obviously increasing the dimensionality of the system makes the structure of attractors more complex and able to generate a larger number of possible behaviors: for instance more multistabilites appear, that can correspond to a larger number of possible memories or choices. In addition, different experimental techniques (fMRI, EEG, etc.) have shown that the non-linear nature of neural dynamics leads to processes of self- organization and phase transitions (Kelso, 1995; Freeman and Vitiello, 2006).

A variety of theoretical models has been used to investigate the properties of large scale networks: it is not possible here to give a detailed review, but they can be subdivided roughly in models derived by the theory of dynamical systems and models derived by analogies with physical systems.

A natural application of the theory of dynamical systems is the concept of neural field, which represents the organization of the cortex with spatially structured neural network whose dynamics are modeled by differential equations in the continuum limit: activity of neural fields can form dynamic spatio-temporal patterns, similar to the spatial distributions experimentally observed in the brain (Wilson and Cowan, 1972; Deco et al., 2008; Bressloff, 2012).

Other models are derived by analogies with physical systems and use typical methods of statistical mechanics: for instance in Ingber (1981) collective neural activities in the cortex are formulated by considering first the microscopical level of synaptic interactions and averaging them spatially to form a mesoscopical domain. The same procedure is then repeated to produce macroscopic spatial-temporal regions, described by the formalism of stochastic processes. A different, but related, approach (Freeman and Vitiello, 2006) utilizes many-body field dynamics, to derive equations describing ordered pattern formation and phase transitions.

More recently the idea has been put forward that analysis of self organized criticality can provide useful insight in the analysis and function of perceptual, cognitive, and motor networks (Parker and Srivastava, 2013) in that these processes offer a way out from the stability-plasticity dilemma (Abraham and Robins, 2005), namely the opposite requirements of stability and plasticity. Self-organized criticality is a feature of non-linear dynamical systems where the macroscopic behavior of a system emerges from the interactions of its component parts. This results in non-equilibrium phase transitions, i.e., sharp variation of neural activity, which depends on the intrinsic dynamics of the system rather than on external inputs (Parker and Srivastava, 2013).

Neural field and physics based models assume that brain states, and hence behavior, arises from activity propagating from microscopic to mesoscopic and finally to macroscopic scale, and these are the basic levels of explanation.

### 2.3. The role of connections

We have seen that large populations of neurons give rise to a rich variety of behavior. The increased dimensionality of the system leads to the appearance of new topological properties and two principles seem to be at work: segregation and integration. Segregation results in the subdivision of the brain in areas which, for instance, respond to specific sensory inputs or perform specialized tasks.

Next, integration among areas is required to process information coming from different sources in the external world and to produce an appropriate behavioral response (Sporns, 2012).

These dual aspects also provide a structural and functional basis to model brain function. Segregation allows more abstract levels of explanation, in that neurons belonging to the same area can be treated as a single variable, for instance by making use of mean field approximation; any description of the brain activity aiming to explain behavior, however, cannot help but taking into account the topology of the connections at the basis of integration among areas (Geary, 2005; Sporns, 2012).

The increased complexity of neural connections in large populations of neurons suggests that the expansion of the range of possible dynamical states does not depend simply on the number of neurons but also on the more complex interactions occurring within the populations. For instance, consider a set of neurons connected in a purely feedforward way. In this case no oscillatory behavior can arise in response to a constant stimulus, but, on the contrary, a feedback loop may give rise to persistent oscillations.

The relevance of the complexity of connections is apparent in the organization of the visual system, where their topology determines the receptive fields from the retina to simple, complex, and hypercomplex cells in the visual cortex, in that the shapes of these receptive fields require excitatory and inhibitory connections to form a precise configuration (Wandell, 1995). Specific structures also characterize the separate, but interactive, visual systems which preside, respectively, to the formation of internal models of the external world, and to the control of object-directed actions (Goodale and Humphrey, 1998).

Even the generation of simple motions requires specific constraints: the neural population must be subdivided in subnets, each able to perform a specific sub-task, connected in a precise way to ensure an ordered succession of neural events. The simplest gesture requires the coordination of activity of different subnets each firing in a precise sequence: more generally motion routines result from the synchronization of the activity of many oscillators, each composed of several neurons, whose phases must have fixed differences to ensure a proper coordination of single steps (Murray, 2002).

The type and topology of connections appear then to play a crucial role in the functions of brain areas at the macroscopical level. As it could be expected this is true also at the lower scale of activity patterns: for instance spatially localized areas of activity can arise from constant input solely if neurons of the area are linked by mutually excitatory and inhibitory synapses forming the so called “Mexican hat” weights distribution (Murray, 2002; Bressloff, 2012). Also, it has been shown that dominant patterns of spontaneous activities in the brain are determined by neural connectivity (Galán, 2008).

Data from fRMI studies on spontaneous brain activity provide further evidence on the role of neural connections. In recent years several studies found that spatiotemporal activity patterns are both complex and consistent across different subjects at rest (Raichle et al., 2001). This evidence poses the question of their origin, namely whether they are the expression of a common cognitive state or the consequence of the constraints imposed by neural connections (Deco et al., 2009).

Several models have been used to predict experimental patterns of activity using connectivity data derived from neuroanatomy. These models represent brain areas as nodes of a graph whose link were derived from neuronatomical data by application of diffusion tensor imaging (DTI). Dynamics of activity of the nodes (brain areas) are simulated, for each model, by a set of differential equations with different variables: membrane potential (Honey et al., 2007; Ghosh et al., 2008), the mean level of spike rate of a neural population (Deco et al., 2009), the phase synchrony of neural oscillations (Cabral et al., 2013). Finally activity can be modeled by a simplified stochastic spin model (Deco et al., 2012).

For each model the correlation between activity of the pairs of nodes (functional connectivity) has been calculated and compared with the correlation between brain areas. The results show, for all models, similar predictions and good agreement between the experimental and simulated correlations (Cabral et al., 2013).

Two conclusions can be drawn from these analyses. First, observed spatiotemporal activity patterns in resting state can be derived just as a consequence of constraints imposed by neural connections among brain regions. Next, note that the models differ by the type of variables and the only common feature is the connectivity matrix, and yet their results are similar. These results, then, support the idea that connectivity is central in the formation of patterns of activity in the brain.

Such idea is at the core of the Connectome project (Bullmore and Sporns, 2009; Sporns, 2012) that intends to understand the complete details of neural connectivity and to construct a map of the complete structural and functional neural connections *in vivo* (Sporns et al., 2005; Hagmann et al., 2007, 2008).

## 3. Discussion

It is often said that the human brain is the most complex structure in the known universe, even though how such complexity can be computed is still a open question. In Tononi et al. (1994), complexity is derived by measures of mutual information, but other definitions could be considered, based on the entropy of the states of neural populations (Shiner et al., 1999). In any case the complex nature of the brain reveals itself in the structure of its connections and patterns of activities. These two aspects are inextricably linked: the structure of interactions among elements of a neural population generates patterns of activity of increasing complexity.

If the single neuron can just perform a scale transformation of the inputs, pairs of mutually connected neuron can give rise to a variety activity patterns, characterized by the presence of attractors and sustained oscillations. These patterns result from the constraints that weights impose on activities. Also, we have shown that in large neural systems the processes of integration and segregation of connections give rise to a greater variety of activities of neurons and neuron groups.

As mentioned earlier, in models of biological networks events are usually supposed to occur at three canonical scales, namely: microscopic, mesoscopic, and macroscopic, to which correspond different levels of explanation.

Inside each scale some finer subdivision can be considered. For instance, motifs, small repetitive networks occurring in large neural populations and supposed to be building blocks of larger networks (Sporns and Kötter, 2004; Battaglia et al., 2012) can be thought of as an intermediate level between microscopic and mesoscopic scales. Also networks devoted to specific behavioral or cognitive tasks can provide a link between mesoscopic and macroscopic levels.

An interesting suggestion has been presented in West and Deering (1994): in many physical systems “*exists a critical dimension above which fluctuations have only a quantitative effect, but below which the fluctuation can be amplified to modify the qualitative behavior of the phenomenon*.”

In the context of neurobiology, this observation could be translated to mean that domains in the cortex in which variations of activity are amplified into sharp transitions implicitly determine a proper scale for the explanation, for instance, of the sensory or cognitive responses to an input.

The focus of the present work is on the connectivity among neurons in large neural populations and considers a simple neuron model with complex connections, so it can be thought of as situated close to one end of the conceptual space proposed in Cessac and Samuelides (2007); moving across this space one can find models with different emphasis on the neuron/connectivity relationships. At the opposite end of the spectrum with respect to the approach presented here is the analysis of the computational properties of the single neuron, which appears to be able to perform also complex computations (Rieke, 1999; Dayan and Abbott, 2001; Koch, 2004). Each specific model can be backed (or disproved) by specific types of data, from recording of electrical activity for single neurons or small networks to activity maps, for instance obtained with fMRI techniques, for large populations.

### Conflict of interest statement

The authors declare that the research was conducted in the absence of any commercial or financial relationships that could be construed as a potential conflict of interest.
